# *Mycobacterium tuberculosis* Modulates miR-106b-5p to Control Cathepsin S Expression Resulting in Higher Pathogen Survival and Poor T-Cell Activation

**DOI:** 10.3389/fimmu.2017.01819

**Published:** 2017-12-18

**Authors:** David Pires, Elliott M. Bernard, João Palma Pombo, Nuno Carmo, Catarina Fialho, Maximiliano Gabriel Gutierrez, Paulo Bettencourt, Elsa Anes

**Affiliations:** ^1^Host-Pathogen Interactions Unit, Faculty of Pharmacy, Research Institute for Medicines, iMed-ULisboa, Universidade de Lisboa, Lisboa, Portugal; ^2^Host-Pathogen Interactions in Tuberculosis Laboratory, The Francis Crick Institute, London, United Kingdom

**Keywords:** tuberculosis, host-directed therapies, microRNAs, cathepsin S, antigen presentation, lysosomal enzymes

## Abstract

The success of tuberculosis (TB) bacillus, *Mycobacterium tuberculosis* (Mtb), relies on the ability to survive in host cells and escape to immune surveillance and activation. We recently demonstrated that Mtb manipulation of host lysosomal cathepsins in macrophages leads to decreased enzymatic activity and pathogen survival. In addition, while searching for microRNAs (miRNAs) involved in posttranscriptional gene regulation during mycobacteria infection of human macrophages, we found that selected miRNAs such as miR-106b-5p were specifically upregulated by pathogenic mycobacteria. Here, we show that miR-106b-5p is actively manipulated by Mtb to ensure its survival in macrophages. Using an *in silico* prediction approach, we identified miR-106b-5p with a potential binding to the 3′-untranslated region of cathepsin S (CtsS) mRNA. We demonstrated by luminescence-based methods that miR-106b-5p indeed targets CTSS mRNA resulting in protein translation silencing. Moreover, miR-106b-5p gain-of-function experiments lead to a decreased CtsS expression favoring Mtb intracellular survival. By contrast, miR-106b-5p loss-of-function in infected cells was concomitant with increased CtsS expression, with significant intracellular killing of Mtb and T-cell activation. Modulation of miR-106b-5p did not impact necrosis, apoptosis or autophagy arguing that miR-106b-5p directly targeted CtsS expression as a way for Mtb to avoid exposure to degradative enzymes in the endocytic pathway. Altogether, our data suggest that manipulation of miR-106b-5p as a potential target for host-directed therapy for Mtb infection.

## Introduction

*Mycobacterium tuberculosis* (Mtb) has succeeded in infecting about one third of the human population by evading innate and adaptive immune responses. Approximately 10% of chronically infected people will manifest the tuberculosis (TB) disease as age, HIV and other illnesses compromise their immune systems (1). From the past 30 years, the greatest threat evolving from this infection was the emergence of drug-resistant strains resulting in multidrug-resistant TB, which accounts for approximately 4% of the new cases of disease. This lead us to search for host targets that may be manipulated during infection to boost the immune responses blocked by Mtb as alternative therapeutic to current antimicrobials.

One of the hallmarks of Mtb pathogenesis is the ability to persist in host professional phagocytes such as macrophages (Mø) (2). This persistence is enabled by the control of host cells functions, particularly those involved in phagosome maturation and fusion with lysosomes (2–5). Usually these processes lead to the acidification of the phagosome and activation of lysosomal enzymes such as proteases and lipases but during Mtb infection these events are halted and the bacteria persist in a less degradative environment (3, 6). An important group of degradative lysosomal enzymes for bacterial clearance includes cysteine cathepsin proteases. Cathepsin activity is optimized for the low pH of the lysosome, which helps to restrict it to this compartment under normal conditions. They are integrated into most processes associated with the lysosome as protein processing or degradation, autophagy, direct pathogen killing, antigen presentation, cellular stress signaling and lysosome-mediated cell death (7). These enzymes can also be secreted, in some circumstances which results in the degradation of extracellular targets such as ECM and granulomas (8).

However, it is yet to decipher how Mtb interferes with cathepsins (Cts) regulation and activity. We recently demonstrated that some Cts are downregulated during Mtb infection and that Mtb controls cathepsins and their inhibitors cystatins at the level of gene expression and proteolytic activity (9). Among the most differentially regulated cathepsins during Mtb infection when compared to *Mycobacterium smegmatis*, a non-pathogenic mycobacteria that is cleared within Mø, was CtsS. CtsS possesses a broad pH profile of activity and the maintenance of a significant endoproteolytic activity at neutral pH, suggesting that this enzyme may have important function along all endocytic pathway (10). Accordingly, our previous results indicated that pathogenic mycobacteria could be killed to some extent in early phagosomes within a pH 6.4–5.5 environment (6). CtsS constitutive expression is restricted primarily to antigen presenting cells and its mRNA expression and protein levels are upregulated by IFNγ [as their promoter responds to interferon-related factor 1 (IRF-1) transcription factor] (11). We found that in IFNγ-activated Mø, CtsS gene expression was highly inhibited during Mtb infection when comparing to non-infected cells (9). In addition, IRF-1 responding promoter and therefore CtsS, influence several important biological processes, including maturation and trafficking of MHC class II molecules required for antigen presentation (12, 13). Mtb has indeed ability to block antigen presentation by affecting the intracellular trafficking of MHC class II molecules *via* cathepsin S (CtsS) (14–16). Moreover, inhibition of CtsS activity has been implicated in autophagy (17) and IFN-γ induces autophagy during mycobacteria infection (18) leading to increased intracellular bacterial killing.

To address how Mtb manipulates CtsS downregulation and, in one attempt to restore the control over CtsS in Mø infected with Mtb, we investigated the potential involvement of microRNAs (miRNAs). miRNAs are predictable intermediates in this process due to their wide regulatory role. These molecules are small, non-coding regulator RNAs with 19–22 nucleotides long that are involved in posttranscriptional gene expression control (19). They silence their targets expression by forming complexes with the RNA-induced silencing complex and then binding their 7 nucleotide-long “seed region” with a complementary region of mRNA, leading to termination of translation and/or mRNA degradation (20, 21). There are already several studies proposing modulation of miRNAs by mycobacteria in order to increase the success of their infection by impairing the release of proinflammatory cytokines (22), controlling phagocytosis (23), preventing cell recruitment to the locus of infection (24) and miRNAs are also proposed as biomarkers of TB disease [recently reviewed by Ref. (25)].

Here, we show miR-106b-5p isoform as another miRNA strongly upregulated during Mtb infection in contrast to challenge with non-pathogenic *M. smegmatis*. Using *in silico* prediction approach, we found that miR-106b-5p has a high probability of binding to CtsS mRNA. Interestingly, miR-106b was described as the posttranscriptional target of the autophagy-related gene 16L1 (ATG16L1) regulating autophagy in the context of epithelial cells and Crohn’s disease (26). Our data indicates that the consequence of miR-106b-5p manipulation by Mtb is a decrease of CtsS activity concomitant with an increase of the intracellular survival of the bacteria and decreased human leukocyte antigen (HLA)-DR class II surface expression in human macrophages, similar to what occurs during siRNA silencing of CtsS. Interestingly, the function of miR-106b-5p on CtsS activity in the context of infection was independent of autophagy. By using inhibitors of this miR, we were able to overcome the Mtb manipulation of the Mø anti-mycobacterial activity. Altogether, our data suggest that manipulation of miR-106b-5p as a potential target for host-directed therapy (HDT) for Mtb infection.

## Materials and Methods

### Cell Lines and Culture Conditions

Human monocyte-derived Mø and CD4 lymphocytes were obtained by, respectively, isolating CD14^+^ monocytes and CD4^+^ lymphocytes from buffy coats of healthy PPD^+^ blood donors provided by the national blood institute (Instituto Português do Sangue e da Transplantação, Lisbon, Portugal) following a protocol established between Dr. Anes and the Portuguese Institute for Blood, allowing access to buffy coats from healthy blood donors, for scientific research with academic purposes only. No personal details from the donors were provided by the supplier. The cells were separated using MACS cell separation system (Miltenyi Biotec). Differentiation of the monocytes into macrophages proceeded as previously described (27). When required, Mø were stimulated with 100 IU/ml IFNγ, overnight, prior to infection.

### Bacterial Cultures

The strain *M. smegmatis* mc^2^155, containing a p19 (long lived) EGFP plasmid was kindly provided by Dr. Douglas Young (The Francis Crick Institute, London, UK), and the green fluorescent protein-expressing strain of Mtb (H37Rv-pEGFP) plasmid was a kind gift from G. R. Stewart (University of Surrey, United Kingdom). *M. smegmatis* was grown in medium containing Middlebrook’s 7H9 Medium (Difco), nutrient broth (Difco) supplemented with 0.5% glucose and 0.05% tyloxapol at 37°C on a shaker at 200 rpm (23). Mtb H37Rv was grown in Middlebrook’s 7H9 medium and supplemented with 10% OADC enrichment (Difco) (28). All experimental procedures using live Mtb were performed in the Biosafety Level 3 laboratory at the Faculty of Pharmacy of the University of Lisbon, respecting the national and European academic containment level 3, laboratory management and biosecurity standards, based on applicable EU Directives. All procedures have been approved by the faculty’s biological safety committee.

### Infection of Macrophages

Bacterial cultures on exponential grown phase were centrifuged, washed in phosphate-buffered saline (PBS). Bacteria were then resuspended in the desired culture medium without antibiotics. In order to dismantle bacterial clumps, the bacterial suspension was subjected to 5 min of ultrasonic bath. Residual clumps were removed by 1 min centrifugation at 500 *g*. Single-cell suspension was verified by fluorescence microscopy. Macrophages were infected with an MOI of 1 for 3 h at 37°C with 5% CO_2_. Following internalization, cells were washed with PBS and resuspended in appropriate culture medium without antibiotics.

### RT-qPCR

Mø were seeded in six-well plates at a density of 2 × 10^6^ cells per well. RNA was isolated and purified from infected cells using Trizol reagent (Invitrogen) and following the manufacturer’s protocol. The relative quantification of miRNAs in total RNA samples was performed by Exiqon (DK) miRNA qPCR services on RNA purified samples in triplicate. The specific quantification of miR-106b-5p in total RNA samples was performed in our laboratory using miRCURY LNA™ Universal RT miRNA PCR system (Exiqon) according to the manufacturer protocol and using the Exiqon LNA™ PCR primer sets: hsa-miR-106b-5p (205884), hsa-miR-23a-3p (204772), hsa-miR-23b-3p (204790), and hsa-miR-24-3p (204260). The qPCR was performed using an ABI 7300 Real Time PCR. The reaction proceeded as follows: 1 cycle of 95°C for 10 min, followed by 40 cycles of 95°C for 10 s and 60°C for 1 min. The miRNA expression profiles were normalized to the average obtained between miR-23a, miR-23b, and miR-24, whose expression levels were stable under the experimental conditions applied in this study (23).

### Transfection

Transfection with anti-CtsS siRNA or with miR-106b-5p mimics and inhibitors was performed with Biontex K2^®^ Transfection System. Macrophages were first incubated for 2 h with 4 µl/ml of K2 Multiplier reagent in culture medium. Then, they were incubated for 24 h with the transfection reagent and 100 nM of SMARTpool ON-TARGETplus human CTSS siRNA or with miRIDIAN miRNA human hsa-miR-106b-5p mimics or hairpin inhibitors and the respective siRNA or miRNA non-targeting controls (GE Dharmacon) in a ratio of 5 μl_reagent_:1 μg_siRNA_ in antibiotic-free medium. Following that, transfection medium was removed and the cells were incubated for 3 days in fresh medium prior to any experiment in order to achieve maximum silencing. The transfection efficiency achieved was approximately 95%, as evaluated by flow cytometry using siGLO Green Transfection Indicator (GE Dharmacon).

### miR-106b-5p Target Validation

A 413 bp fragment of the 3′-untranslated region (3′-UTR) of the human CtsS gene (CTSS) containing a sequence complementary to the seed region of miR-106b-5p, was amplified by PCR using the Phusion^®^ Hot Start II DNA Polymerase (New England BioLabs^®^, MA, USA), following the manufacturer’s instructions (forward primer: 5′-GCGAGCTCCAAGAAATATGAAGCACTTTCTC-3′, reverse primer: 5′-CCCTCGAGTTTTTTGAAACAGAGTCTCCACT-3′). The fragment was inserted into the pmirGLO Dual Luciferase miRNA Target Expression Vector (Promega Corporation), between the *Sac*I and *Xho*I restriction sites, to originate a recombinant plasmid expressing the 3′-UTR fragment of the human CtsS gene. Mutant plasmids were constructed bearing a single point mutation on the primer that includes the seed sequence, thus incorporating this mutation on the amplicon during the PCR amplification. The forward primer 5′-GCGAGCTCCAAGAAATATGAAGCATTTTCTC-3′ for CTSS 3′-UTR plasmid was used. Pointmutation is underlined. All restriction enzymes and the DNA ligase used were from New England BioLabs^®^. PCR products and restriction products were purified using the illustra™ GFX™ PCR DNA and Gel Band Purification Kit (GE Healthcare). Recombinant plasmids were stored and propagated inside JM109 *E. coli* cells. The constructed plasmid, miR-106b-5p mimics and miRNA negative controls were transfected into HEK 293 T-cells using ScreenFect^®^A Transfection Reagent (ScreenFect GmbH), following the manufacturer’s instructions. After transfection, the dual luciferase assay was initiated using the Dual-Luciferase^®^ Reporter (DLR™) Assay System kit (Promega Corporation), following all the manufacturer’s instructions.

### Western Blotting

Mø were seeded in six-well plates at a density of 2 × 10^6^ cells per well. Total proteins were recovered with 200 µl of Laemmli buffer (Sigma-Aldrich). Protein extracts were subjected to electrophoresis in 12% SDS-PAGE gels, transferred to a nitrocellulose membrane and blocked in Tris-buffered saline with 0.1% Tween 20, 5% of bovine serum albumin (BSA). The nitrocellulose membranes were incubated with primary antibodies specific for human CtsS and β-tubulin (Abcam, 92780 and 6046, respectively), overnight at 4°C. All membranes were washed and incubated with secondary HRP-conjugated antibodies (Biorad). The bands were visualized with Luminata Crescendo Western HRP substrate (Merck Millipore) and quantified using ImageJ (29).

### Enzymatic Activity

Mø were seeded in six-well plates at a density of 2 × 10^6^ cells per well, and were recovered with a 5 mM EDTA/PBS solution. Cell lysis and measurement of the enzymatic activity was performed using the Cathepsin Activity Fluorometric Assay kits (Biovision) specific for each cathepsin and following the manufacturer’s instructions. Essay specificity was verified by treating the cell lysates with specific inhibitors for each cathepsin, provided in the kit. The fluorescence intensity was measured by the Tecan M200 spectrofluorometer.

### Immunofluorescence

Mø were seeded in 24-well plates at a density of 3 × 10^5^ cells per well. Following the experiments, the samples were fixed with 4% paraformaldehyde in PBS for 1 h and quenched by incubating with PBS 50 mM NH_4_Cl. Macrophages were permeabilized with 0.1% Triton in PBS for 5 min and subsequently washed and blocked with 1% BSA in PBS. Cells were stained with anti-LC3b antibody (Cell Signaling, 2775) in 1% BSA/PBS overnight at room temperature. Following that, the cell nuclei were stained with 5 µg/ml of hoechst dye (Thermo Scientific) for 10 min. Samples were mounted with ProLong™ Gold antifade mountant and analyzed by confocal microscopy (Leica AOBS SP5).

### Flow Cytometry

Mø were seeded in 24-well plates at a density of 3 × 10^5^ cells per well. Following the experiment, macrophages were recovered with 5 mM EDTA/PBS solution and fixed with 4% paraformaldehyde for 1 h. Following that, cells were stained for 30 min with antibodies specific for human HLA-DR (clone L243, Biolegend), CD14, CD4 (BD Biosciences), or Annexin V and propidium iodide (Immonotools, GmbH) for the quantification of apoptotic and necrotic cells. Samples were analyzed in Guava easyCyte™ 5HT flow cytometer.

### Assays for CD4 Proliferation

Mø were seeded in 48-well plates at a density of 1.5 × 10^5^ cells per well. Following 24 h of infection CD4 lymphocytes were added to the culture at a ratio of 5 lymphocytes per Mø. CD4 lymphocytes were recovered after 5 days of coculture and quantified using Guava easyCyte™ 5HT flow cytometer.

### Assays for Bacteria Intracellular Survival

Mø were seeded in 96-well plates at a density of 5 × 10^4^ cells per well. When required, infected cells were lysed in 0.05% Igepal solution. Serial dilutions of the resulting bacterial suspension were plated in Middlebrook 7H10 with 10% OADC (Difco) and incubated for 2–3 weeks at 37°C before colonies were observable.

### Statistical Analysis

Data are presented as mean ± SE except if stated otherwise. Statistical analysis was made using SigmaPlot 12. Multiple group comparisons were made using ANOVA one parameter tests followed by pairwise comparisons of the groups using Holm-Sidak test. Two group comparisons were made using Student’s *t*-test. All the prerequisites of the tests were verified. The considered nominal alpha criterion level was 0.05 below which differences between samples were deemed significant.

## Results

### Mtb Induces the Expression of miR-106b-5p in Human Mø

**Figure 1:**
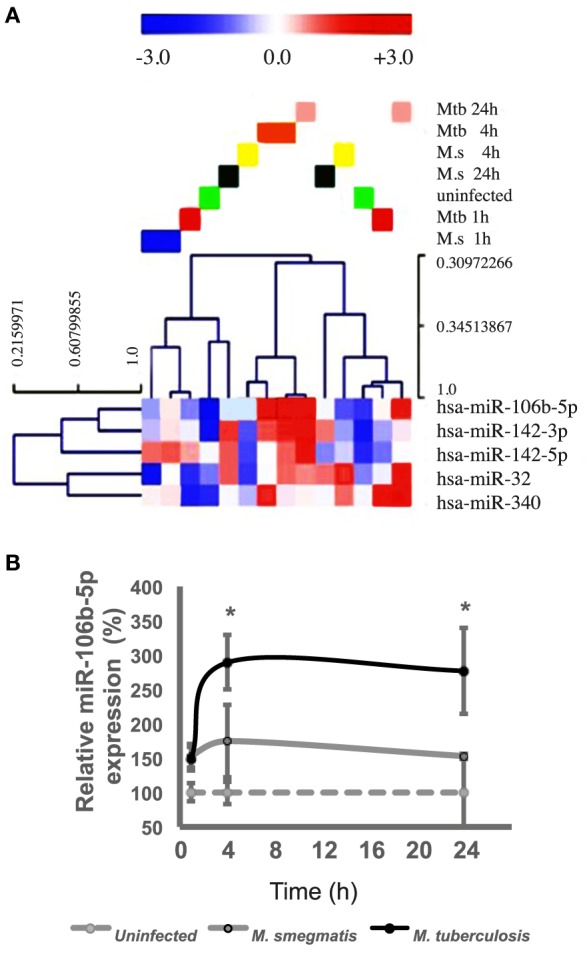
miR-106b-5p is upregulated in human Mø during *Mycobacterium tuberculosis* (Mtb) infection. **(A)** Heat Map of human microRNA (miRNA) expression in Mø infected with *M. tuberculosis* and *Mycobacterium smegmatis* and Unsupervised Hierarchical Clustering performed by Exiqon. The clustering is performed on all samples and on the miRNAs detected in all samples. Each row represents one microRNA and each column represents one sample. The miRNA clustering tree is shown on the left. The color scale shown illustrates the relative expression level of a miRNA across all samples: red color represents an expression level above mean, blue color represents expression lower than the mean. The normalized (dCp) values have been used for the analysis. Normalizer microRNAs have been omitted from the analysis. **(B)** miR-106b-5p quantification by qt-RT-PCR along 48 h of infection. Values are relative to uninfected control cells and represent means of three biological replicates while error bars show the SE. Asterisks indicate statistical significance between samples (*P < 0.01).

We previously performed studies to assess the involvement of miRNAs in posttranscriptional regulation of phagocytosis in the context of a murine model of mycobacterial infection (23). A global transcriptomic analysis of Mø infected with *M. smegmatis*, revealed among the most differentially regulated miRNAs miR-142-3p; -5p; miR-32 (upregulated) and miR-106b-5p (downregulated). Here, we asked what would be the effect of human Mø in response to the infection of a pathogenic species, Mtb relatively to the non-virulent *M. smegmatis*, a species that is cleared in these phagocytic cells. Human primary Mø derived from blood monocytes of healthy donors were infected either with *M. smegmatis* or with Mtb and RNA samples were extracted for RT-qPCR analysis at 1, 4, and 24 h postinfection (p.i.). The Exiqon microarrays heat map generated diagram (Figure 1A) shows the result of the two-way hierarchical clustering of miRNAs and samples. Each row represents one miRNA and each column represents one sample. The miRNA clustering tree is shown on the left. The color scale shown at the bottom illustrates the relative expression level of a miRNA across all samples: red color represents an expression level above mean, blue color represents expression lower than the mean. miRNAs found common to all infection conditions are displayed. Notably, the results showed miR-106b-5p strongly upregulated during Mtb infection (Figure 1A). Our previous results indicated that miR-106b-5p was downregulated early upon *M. smegmatis* infection of the mouse Mø cell line J774 (23) while in the present study, using human cells, no effect was observed. These results emphasize the influence of the host cell species for the response to infection *in vitro*. Given these differences, we decided to use human primary Mø in our experiments to assess the response to Mtb infection.

For an accurate quantification of gene expression and in order to confirm that miR-106b-5p is being differentially regulated during infection with Mtb relatively to *M. smegmatis*, we independently validated the RT-qPCR analysis. Our results confirm those performed by Exiqon, i.e., a distinct phenotype between the two species (Figure 1B), with *M. smegmatis* infection having no effect in miR-106b-5p expression while the challenge with Mtb led to a 2-fold increase in miR-106b-5p RNA at 4 h which was maintained for the 24 h assay. Thus, Mtb infection upregulates miR-106b-5p in primary human Mø (Figure 1).

### miR-106b-5p Targets CtsS mRNA

Using target prediction tools based on the miRanda (30), miRtaget2 (31), and miRmap (32) algorithms, we have identified several potential targets of miR-106b-5p, including CtsS mRNA (CTSS). Conversely, we then asked how many miRNAs are predicted to target CTSS. From the 1,054 potential targets generated by the miRmap algorithm the top 30 with highest score are shown in Table 1 with miR-106b-5p at the 24th position with a score of 90.35. These results, combined with previous data from our group, establishing an important role of CtsS in Mtb infection (9), led us to generate the hypothesis of whether CtsS was a real target of miR-106b-5p, and if it has implications in bacterial survival, cell death as well as antigen presentation.

**Table 1:** Top 30 microRNAs predicted to bind to CTSS.

Rank	miRNA	Probability exact	miRmap score
1	hsa-miR-520c-3p	98.74	90.76
2	hsa-miR-520b	98.74	90.76
3	hsa-miR-520e	98.74	90.76
4	hsa-miR-520d-3p	98.74	90.73
5	hsa-miR-302c-3p	98.74	90.73
6	hsa-miR-302a-3p	98.74	90.73
7	hsa-miR-520a-3p	98.74	90.69
8	hsa-miR-373-3p	98.74	90.69
9	hsa-miR-526b-3p	99.00	90.67
10	hsa-miR-372	98.74	90.65
11	hsa-miR-302b-3p	98.74	90.63
12	hsa-miR-302d-3p	98.74	90.62
13	hsa-miR-302e	98.74	90.58
14	hsa-miR-93-5p	99.00	90.57
15	hsa-miR-586	97.13	90.51
16	hsa-miR-519d	99.00	90.50
17	hsa-miR-17-5p	99.00	90.49
18	hsa-miR-5787	99.74	90.49
19	hsa-miR-378g	99.78	90.49
20	hsa-miR-20b-5p	99.00	90.45
21	hsa-miR-106a-5p	99.00	90.44
22	hsa-miR-4674	99.62	90.43
23	hsa-miR-20a-5p	99.00	90.35
24	hsa-miR-106b-5p	99.00	90.35
25	hsa-miR-612	99.78	90.32
26	hsa-miR-4505	99.74	90.30
27	hsa-miR-651	98.01	90.27
28	hsa-miR-520f	87.32	90.24
29	hsa-miR-3619-5p	94.50	90.22
30	hsa-miR-939-3p	16.73	90.19

*Based on the miRmap algorithm*.

**Figure 2:**
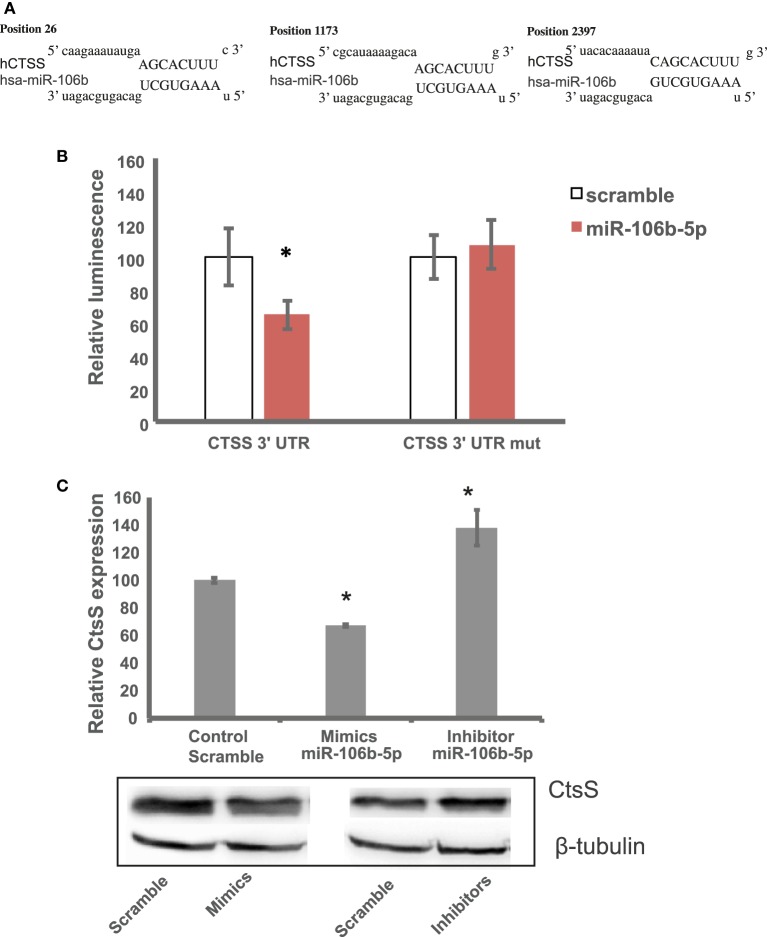
miR-106b targets cathepsin S (CtsS). **(A)** Three different predicted sites were found after screening the 3′-untranslated region (3′-UTR) region of CTSS for potential miR-106b-5p binding sites using RNAhybrid. **(B)** The interaction was confirmed by means of relative luciferase activity measured in HEK 293 T-cell line transfected with CTSS 3′-UTR plasmid or CTSS 3′-UTR mut plasmid in combination with scramble control or miR-106b-5p mimic. Values are relative to the respective scramble control-transfected cells. The columns show means of three biological replicates each measured in triplicates while error bars show the SE. Asterisks indicate statistical significance relative to the respective scramble control-transfected cells (*P < 0.05). **(C)** CtsS protein expression in human Mø transfected with miR-106b-5p mimics or inhibitors. Values are relative to the scramble control and represent means of two biological replicates (*P < 0.001).

After screening the 3′-UTR of CTSS for potential miR-106b-5p interaction using RNAhybrid (33), we identified three different binding sites (Figure 2A). Our initial results portrayed an induction of miRNA-106b-5p in Mtb-infected Mø in agreement with our previous findings that CtsS is downregulated by Mtb infection (9). To confirm this interaction, we used a dual luciferase reporter vector system in which we inserted a fragment of the 3′-UTR sequence for CtsS into the pmirGLO dual-luciferase target expression vector. This fragment included one putative sequence complementary to the “seed region” of miR-106b-5p. Using this system, we were able to assess the specific interaction between the miRNA and the 3′-UTR sequence by analyzing the resulting decrease in luciferase expression in HEK 293 T-cells transfected with both the plasmid and the miRNA. The results showed a significant reduction in luciferase expression (*P* < 0.05, paired *t*-test) during cotransfection with miR-106b-5p and 3′-UTR CTSS plasmid relative to the control (cells transfected with a nonsense miR and the 3′-UTR CTSS reporter plasmid) (Figure 2B). On the contrary, when a plasmid bearing a mutation on the 3′-UTR CTSS seed sequence by one nucleotide substitution was tested, there was no decrease in luciferase activity detected.

Next, we tested whether we were able to modulate CtsS protein levels in non-infected cells by overexpressing or inhibiting miR-106b-5p. For this, we transfected primary human Mø with miR-106b-5p mimics or inhibitors and analyzed protein levels by Western blotting (Figure 2C). The resulted silencing of approximately 40% for CtsS using mimics was comparable to the reduction of luminescence in the luciferase assay. Conversely, when using inhibitors an increase of protein levels of around 40% was observed (Figure 2C). Altogether, the data indicate the specificity of the interaction between CTSS 3′-UTR binding sites and miR-106b-5p leads to CtsS silencing.

### The Modulation of miR-106b-5p Expression Regulates CtsS Protein Amounts in Mtb-Infected Mø

**Figure 3:**
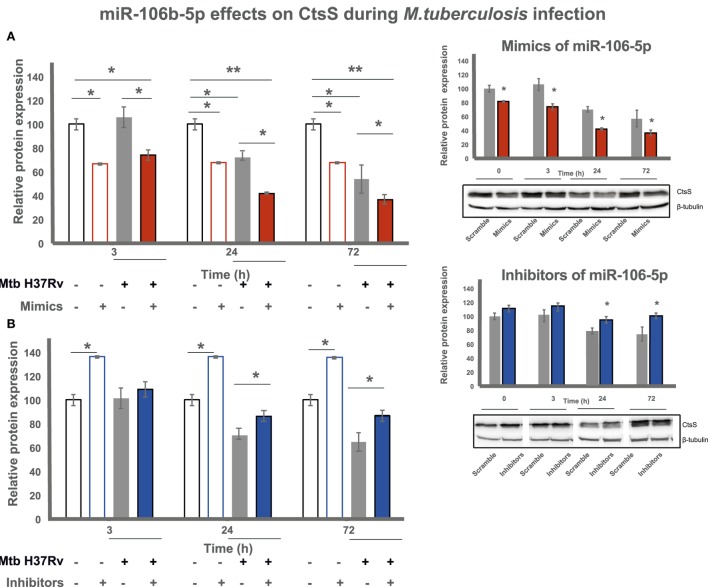
miR-106b-5p regulates cathepsin S (CtsS) during infection with *Mycobacterium tuberculosis*. CtsS protein expression in human Mø transfected with miR-106b-5p mimics **(A)** or inhibitors **(B)** and subsequently infected with Mtb. Proteins were recovered from uninfected Mø (T0) and infected Mø after 3, 24, and 72 h postinfection. The right panels are a different representation of the same data including the protein gel bands (original Wester-blots in Figure S2). Values are relative to uninfected Mø transfected with the respective scramble control and represent means of two biological replicates with error bars depicting the SE. Asterisks indicate statistical significance between indicated samples or relative to the uninfected (T0) controls (*P < 0.05 or **P < 0.001).

Our results showed an early Mtb-dependent upregulation of miR-106-5p and a specific interaction between this miRNA and CTSS. These findings are correlated with our previous results showing that Mtb induces the downregulation of CtsS at 24 h, suggesting that miR-106b-5p might be implicated in this regulation. To further test this hypothesis, we performed similar experiments, as described above, using mimics and inhibitors of miR-106b-5p in the context of Mtb infection. As shown in Figure 3A (right panel), transfecting cells with mimics of miR-106b-5p resulted in a decrease of CtsS protein levels in Mtb-infected cells, at all time points tested, relatively to scramble-infected cells (scramble: infected and transfected with control nonsense RNA). The difference was even more significant when comparing cells treated with mimics after 3 days of infection relatively to control (scramble) non-infected cells (red bars to white bars in the left panel). When the inhibitors of miR-106b-5p were tested, significant effects were observed mostly later in the course of the infection, namely after 24 h p.i. In contrast to miR-106b-5p mimics, the transfection with inhibitors lead to an increase of CtsS levels in infected cells relatively to scramble-infected cells (Figure 3B, right panel). Altogether, miR-106b-5p modulates CtsS expression in human Mø during infection with Mtb.

### miR-106b-5p Modulates the Intracellular Survival of Mtb

**Figure 4:**
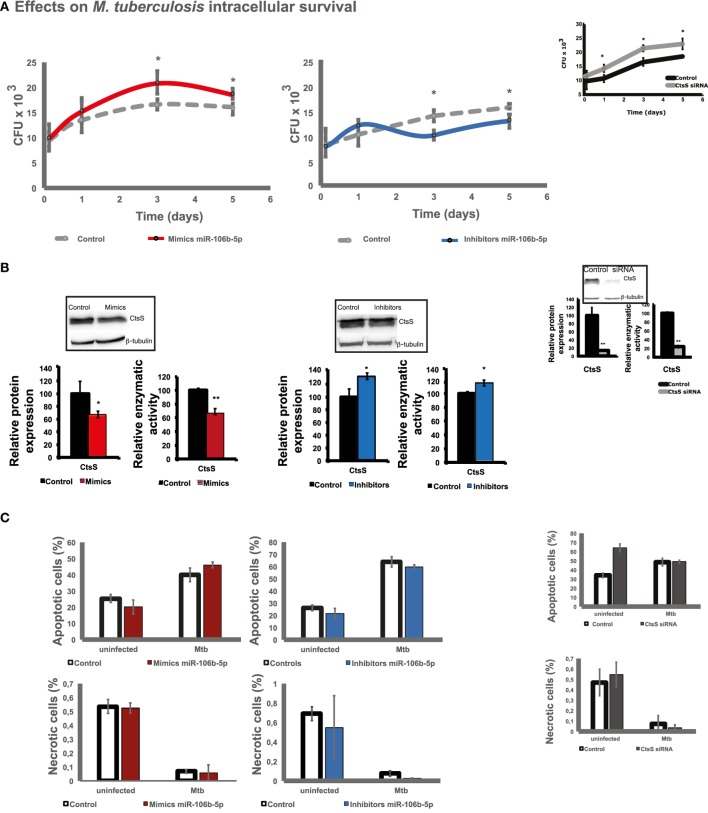

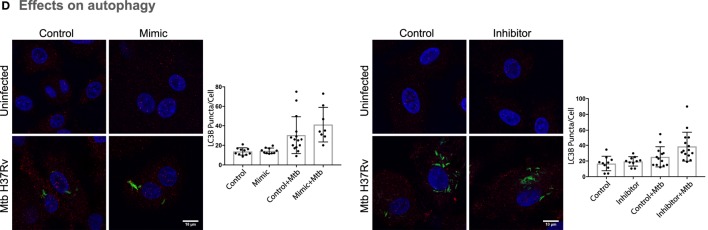
Intracellular survival of *Mycobacterium tuberculosis* (Mtb) in Mø transfected with miR-106b-5p mimics or inhibitors. **(A)** Colony forming units of intracellular bacteria recovered from Mø transfected with miR-106b-5p mimics or inhibitors. CFUs were recovered after 3 h, 1, 3, and 5 days postinfection. Values depict mean colony-forming units representative of three biological replicates measured in duplicate while the error bars depict the SE. Asterisks indicate statistical significance between samples at the same time point (*P < 0.05). **(B)** Effects of mimics or inhibitors on cathepsin S (CtsS) protein levels and enzymatic activity. Values are relative to the scramble control and represent means of two biological replicates (for protein quantification) or means of three biological replicates (for enzymatic activity) (*P < 0.05 or **P < 0.001). **(C)** Effects of mimics or inhibitors on Mø cell death: apoptosis, necrosis. Cell death was measured by flow cytometry after 24 h of infection using fluorescent Annexin V antibodies and propidium iodide (see Figure S1 in Supplementary Material for the respective dot plots). Values show median fluorescence intensity from one representative experiment performed in triplicate while error bars depict SD. **(D)** Effects of mimics or inhibitors on autophagy. LC3 autophagy puncta were observed in uninfected Mø or 24 h postinfection with Mtb by confocal microscopy and quantified using ImageJ. Bar plots represent the mean values of at least eight analyzed microscopy fields (dots) from one representative experiment. Error bars depict the SD.

Given that Mtb manipulates miR-106b-5p expression resulting in reduced CtsS protein levels, we hypothesized that this regulation will affect the endolysosomal enzyme proteolytic activity and subsequently the ability of Mtb to survive inside host cells. To test this, we first analyzed the intracellular survival of Mtb during 5 days of infection in human Mø transfected with mimics or inhibitors. As expected, the mimics for miR-106b-5p gain-of-function, we observed an increase in Mtb survival, relative to the control (scramble transfected and infected cells) at day 3 p.i. (Figure 4A, left panel). In contrast, an exacerbated killing effect from day 3 p.i. was detected by using miR-106b-5p inhibitors in loss-of-function experiments (Figure 4A, right panel). The results were in agreement with a decrease in protein levels and hydrolytic activity for CtsS using mimics (Figure 4B, left panels) and a significant increase in protein and enzyme activity using miR-106-b-5p inhibitors (Figure 4B, right panels) calculated 3 days p.i. A similar trend on Mtb survival using mimics experiments was confirmed by CTSS siRNA (Figure 4A, top right). Altogether, these results show that miR-106b-5p levels can impact intracellular survival of Mtb in human Mø.

To decipher whether the observed miR-106b-5p-dependent pathogen killing/survival was dependent on alternative activation pathways, we tested for the involvement of apoptosis, necrosis or autophagy, during gain- and loss-of-function experiments. We tested whether a decrease on CtsS due to miR-106b-5p gain-of-function will augment apoptosis in infected cells by using Annexin V staining to monitor the event. As shown in Figure 4C and Figure S1 in Supplementary Material, no increase on apoptosis was detected neither in mimic nor in inhibitors transfected Mtb-infected cells relatively to the control as quantified by flow cytometry. We next assessed necrosis also by flow cytometry using propidium iodide-labeled cells and observed no effect on necrosis (Figure 4C-down panels; Figure S1 in Supplementary Material).

Finally, the process of autophagy was examined by immunofluorescence quantification of LC3A/B puncta (Figure 4D). A significant increase in LC3 puncta was observed after infection with Mtb-infected human Mø (Figure 4D). Surprisingly, there were no changes in LC3 puncta when comparing infected cells transfected with miR-106b-5p mimics or with inhibitors relatively to nonsense-scramble-transfected cells (Figure 4D). We concluded that the effect of miR-106b-5p was autophagy independent. Altogether, these results indicate that a knock-down of CtsS in the context of Mtb manipulation of miR-106b-5p is relevant for pathogen intracellular survival by interfering with proteolysis in the endolysomal pathway and independently of autophagy and programed cell death activation.

### miRNA-106b-5p Interferes with the Antigen Presentation Machinery and T-Cell Activation

**Figure 5:**
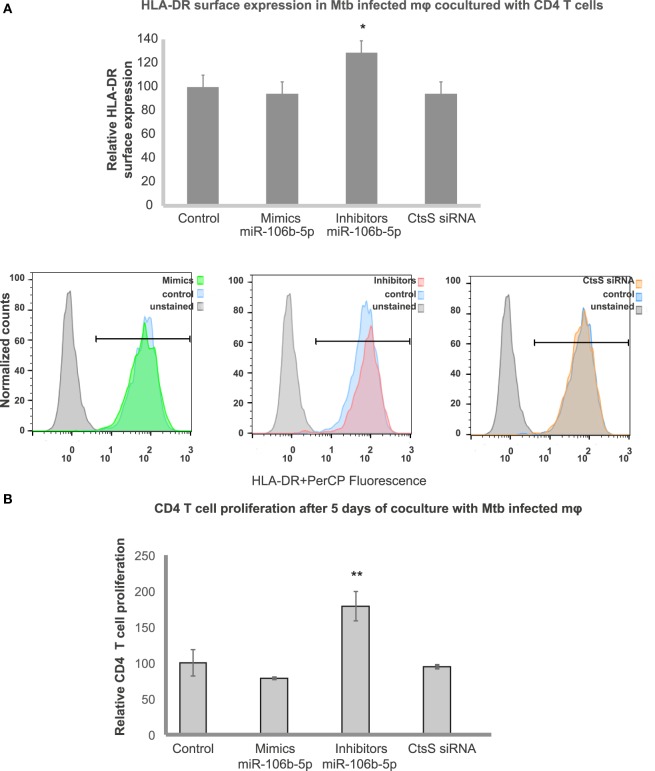
Effects of miR-106b-5p on antigen presentation machinery and T-cell priming. **(A)** Surface expression of human leukocyte antigen (HLA)-DR on Mø transfected with miR-106b-5p mimics, inhibitors or by siRNA for CTSS and infected with *Mycobacterium tuberculosis*. HLA-DR was measured by flow cytometry after 24 h of infection. Values in bar plots represent median fluorescence intensity relative to the respective scramble controls from one representative experiment performed in triplicate while error bars depict SD (*P < 0.05). Raw values from one representative replicate are presented in the fluorescence intensity histograms. **(B)** CD4 T-cell proliferation after 5 days of coculture with Mtb-infected Mø. Following 24 h of the infection with Mtb, CD4 T-cells were added to the infected Mø culture. After 5 days of coculture, the number of CD4 T-cells comparatively to the non-infected controls was determined by flow cytometry. Values in bar plots represent the increase in CD4 T-cell number relative to each uninfected scramble control samples (**P < 0.01).

According to the KEGG pathway database, CTSS is involved in several pathways such as (1) lysosomal, (2) phagosomal, and (3) antigen processing and presentation. CtsS has been implicated in endosomal antigen processing and antigen presentation (13, 34). We then hypothesized that Mtb modulation of miR-106b-5p for CtsS silencing might be linked to poor antigen processing and presentation compromising adaptive immunity response to infection. To test this, we performed miR-106b-5p gain- and loss-of-function experiments in Mtb-infected cells and analyzed changes in the surface expression of HLA-DR class II complexes using flow cytometry (Figure 5). Transfection of infected cells with miR inhibitors led to an increase expression of HLA-DR at the surface of Mø (Figure 5). No differences were observed relatively to HLA-DR surface expression of mimics and CtSS siRNA transfected cells relatively to control cells. Thus, we inferred that Mtb infection silencing of CtsS affect the antigen presentation machinery at a level that no more silencing of the enzyme either by mimics or by siRNA could revert the phenotype. To further evaluate the consequences of antigen presentation dependence on miR-106b-5p modulation, we performed cocultures of treated infected macrophages with CD4 + T-lymphocytes from the same blood samples and evaluated the ability to induce T-cell proliferation (Figure 5B). In order to test this, we used human Mø infected with Mtb from a donor population that is all vaccinated. It is expected in these cells a memory highly effective T-cell state that responds to Mtb infection. Following the same pattern of HLA surface expression, treatment with inhibitors in Mtb-infected cells induces a strong T-cell proliferation relatively to the control, after 5 days post-cocultures as evaluated by flow cytometry (Figure 5B). No changes were detected in all other conditions tested.

## Discussion

This study revealed that miR-106b-5p targets CTSS for protein silencing with consequences in Mtb persistence in human Mø obtained from healthy (BCG vaccinated) donors. Our results indicate that Mtb actively manipulates miR-106b-5p by upregulating their gene expression during infection in opposition to what it is observed with the non-pathogen *M. smegmatis*. We made use of algorithms such as the miRmap to predict miRNA targets based on nucleic acid sequence, probabilistic, thermodynamic, or evolutionary features (32). Based on this and other’s algorithms the mRNA for CtsS was revealed as a potential target. We then asked what would be the most probable miRNAs predicted to bind the 3′-UTR of CTSS. From the 1,054 targets generated by the miRmap algorithm, miRNA-106b-5p showed the 24th highest score (99.35) (Table 1). This score and probability to bind to CTSS is higher than that of miR-3619-5p (99.22), on the 29th position, that has been described and experimentally validated as a miRNA predicted to bind the 3′-UTR of CTSS (35). While both miRs target CTSS, contrary to miR-106b-5p that we found upregulated during Mtb infection, miR-3619-5p was downregulated in BCG-infected THP1 derived Mø. Importantly most members of the miR-17-92 cluster and all members of the miR-17 cluster are present in the list: hsa-miR-17-5p, hsa-miR-20a-5p, hsa-miR-20b-5p, hsa-miR-106a-5p, hsa-miR-106b-5p, and hsa-miR-93-5p which have been implicated all in innate and adaptive immune responses (36). Therefore, we envisage that miR-106b-5p is actively manipulated by Mtb to ensure its survival in innate phagocytic cells and escape to immune surveillance and activation. Furthermore, from the above data we may foresee the relevance of CtsS regulation due to the observed numbers of distinct miRs that are predicted to target this mRNA.

The target was confirmed by luminescence-based methods and the miR-mRNA targeting resulted in about 40% in protein translation silencing. We made use of gain or loss-of-function experiments to modulate miR-106-b-5p during Mtb infection. The results indicated a decrease or increase of CtsS expression, respectively, concomitant with increased Mtb intracellular survival or killing, correspondingly. Altogether, this suggests that CtsS dependent Mtb intracellular survival *via* miR-106b-5p modulation resulted from a hydrolytic activity along the endocytic pathway upon phagocytosis. Mtb is known to block phagosome maturation, preventing its digestion in lysosomes (2–5). However, CtsS is a proteolytic enzyme that as a broad pH for activity along the endocytic pathway and not only into the acidic environment of the phagolysosome (10). Therefore, a modulation of mir-106b-5p to silence CtsS activity may be a pathogen strategy to survive along vesicles in this pathway.

Our results did not reveal significant changes in apoptosis after miR-106b-5p manipulation. Apoptosis has been associated with a mechanism for effective intracellular bacteria clearance (37, 38) therefore the results of miR in Mtb survival could not be attributed to control of apoptosis. Curiously, one of the miRs we showed to be more differentially regulated during mycobacteria infection, in addition to miR-106-b was miR-142-3p (Figure 1A). Both actually targets genes involved in phagocytosis as the endolysosomal enzyme CtsS and the actin binding protein N-WASP, respectively (23). Both are indeed targeting simultaneously the ATG16L1 (39, 40). We and other’s identified miRs relevant during phagocytosis that simultaneously are predicted to manipulate autophagy (26, 35). Some studies suggest that inhibition of CtsS induces autophagy in different cells (17) while for others a silencing of CtsS is likely to affect degradation of autophagosomal contents leading to a block of autophagy in impaired LC3 vesicles (35). Indeed, the knock-down of CtsS was associated with mitophagy dysregulation leading to apoptosis and impairment and accumulation of autophagosomes (41).

Our data argue that the miR-106b-5p-dependent improved control of Mtb is independent of autophagy and more related to the process of phagosomal degradation. Autophagy is an important innate immune intracellular pathway that control mycobacteria in macrophages (18). In the context of CtsS, the downregulation of CtsS along with LAMP1 and CtsD lead to impaired autophagosome–lysosome fusion (42). The fact that miR-106b-5p, in addition to CtsS, also targets ATG16L1 may provide evidence for a double control of autophagy for bacteria survival and persistence. However, it has been recently shown that ATG5 but not ATG16L1 or others autophagy-related genes plays a key role in the host response to mycobacteria (43, 44). Moreover, the inhibition or impairment of ATG16L1 was associated with autophagy block and incapacity to control inflammasome proteolysis in autophagolysosomes resulting in exacerbated IL-1β secretion, inflammation and necrotic cell death in the context of epithelial cells and Crohn’s disease (26, 45).

We neither found an increase on necrosis upon miR-106b-5p mimics or inhibitors treatment that could account for Mtb survival. Other studies associated a IFNγ knock-down leading to autophagy inhibition and therefore a low CtsS content in endolysosomal vesicles to an increase secretion of proinflammatory cytokine IL1β and programmed cell death by necrosis (46). The effect of necrotic programmed cell death as pyroptosis in control bacteria killing is unclear: some data show this process contributes to pathogen killing (47) whereas other data argues that it helps pathogen escaping and spread to non-infected cells (48).

Cathepsin S has been implicated in antigen processing and presentation (12, 13) and that Mtb has indeed ability to block antigen presentation *via* CtsS (14–16). Here, we show that modulation of miR-106b-5p during infection indeed modulates the surface expression of the MHC Class II antigen presentation machinery (HLA-DR). In the case of macrophages infected with Mtb, where HLA-DR antigen presentation was already blocked, the usage of mimics did not change this blockade, similar to what observed during CtsS silencing by conventional siRNA methods (Figure 5B). Importantly, in infected cells treated with inhibitors an increased CtsS activity was concomitant with an increase surface expression of the antigen presentation machinery. This was relevant to establish a bridge for the adaptive immunity as the inhibitors treated infected cells were more able to induce T-CD4 lymphocyte proliferation. A differential regulation of CtsS was already observed in previous published results when comparing the infection of the non-virulent species *M. smegmatis* with macrophage infection with Mtb (9). Indeed, Mtb was shown to induce lower MHC class II expression compared with *M. smegmatis* (49). Here, we show that the manipulation of MHC-II expression through the axis miR-106b-5p/CtsS may overcome the blockade induced during Mtb infection.

A recent study by Meng et al. (50) describes that miR-106b-5p is downregulated in samples from latently infected individuals, where Mtb is known to be not dividing and is enclosed within the phagosomes, avoiding the fusion with lysosomes containing cathepsins and other digestive enzymes. In our experiments, we describe that miR-106b-5p is upregulated in macrophages infected with Mtb which are dividing and producing an infection that mimics active TB, with increasing numbers of CFU overtime (Figure 4A control samples). Therefore, the work of Meng et al., combined with the present study might suggest that the miR-106b-5p is a good candidate to be used as a biomarker to distinguish between active and latent TB, although further experimental evidence would be required to formally validate this hypothesis.

Tuberculosis is a major worldwide public health concern among infectious diseases and the treatment remains a challenge. HDTs represent a new strategy for adjuvant TB therapy trying to potentiating key components of host antimycobacterial effector mechanisms, while restricting inflammation (51, 52). miRNAs are key players in controlling gene expression and are nucleic acids feasible for targeted drug delivery to phagocytic cells (53). The present study reveals that Mtb blocks CtsS *via* miR-106b-5p for preventing innate and adaptive immune responses to persist in the host. We further provide means how to revert this process by manipulation of miR-106-5p helping to control the infection. Our study opens the door for future manipulation of miR-106b-5p to enhance the antimicrobial activity of innate immune cells.

## Author Contributions

Conceptualization: EA, DP, and PB. Methodology, acquisition, and analysis: DP, EB, NC, MG, PB, and EA. Investigation: DP, JP, NC, and CF. Writing: EA and DP. Supervision: EA. Read, commented, and approved the final version of the manuscript: all authors.

## Conflict of Interest Statement

The authors declare that the research was conducted in the absence of any conflict of interest.

## Supplementary Material

Figure S1 Flow Cytometry generated dot-plots for necrosis and apoptosis effects of mimics and inhibitors of miR-106b-5p on *Mycobacterium tuberculosis* (Mtb)-infected cells and on non-infected cells. Cell death was measured by flow cytometry after 24 h of infection using fluorescent Annexin V antibodies and propidium iodide.

Figure S2 Original Western blots from Figure 3. Cathepsin S protein expression in human Mø transfected with miR-106b-5p mimics or inhibitors and subsequently infected with *Mycobacterium tuberculosis* (Mtb). Proteins were recovered from uninfected Mø (T0) and infected Mø after 3, 24, and 72 h postinfection.
