# Supplementary material for: Mycobacterium tuberculosis Modulates miR-106b-5p to Control Cathepsin S Expression Resulting in Higher Pathogen Survival and Poor T-Cell Activation
Source: Front Immunol. 2017 Dec 18;8:1819. doi: 10.3389/fimmu.2017.01819 (PMC5741618; doi:10.3389/fimmu.2017.01819)
Supplement: Figure S1 — Flow Cytometry generated dot-plots for necrosis and apoptosis effects of mimics and inhibitors of miR-106b-5p on Mycobacterium tuberculosis (Mtb)-infected cells and on non-infected cells. Cell death was measured by flow cytometry after 24 h of infection using fluorescent Annexin V antibodies and propidium iodide. [file Presentation_1.PDF]

Supplementary Information

Mycobacterium tuberculosis modulates miR-106b-5p to control Cathepsin S expression resulting in higher pathogen survival and poor T cell activation

David Pires[1,&], Elliott Bernard[2], João Palma Pombo[1], Nuno Carmo[1], Catarina Fialho[1], Maximiliano Gabriel Gutierrez[2], Paulo Bettencourt[1,#,&] and Elsa Anes[1,*]

[1]Host-Pathogen Interactions Unit, Research Institute for Medicines, iMed-ULisboa, Faculty of Pharmacy, Universidade de Lisboa, Portugal.

[2]Host-Pathogen Interactions in Tuberculosis Laboratory, The Francis Crick Institute, 1 Midland Road, London, NW1 1AT United Kingdom

*Correspondence:

Elsa Anes

eanes@ff.ulisboa.pt.

& Contributed equally

#Present address: The Jenner Institute, University of Oxford, United Kingdom.

Figure S1

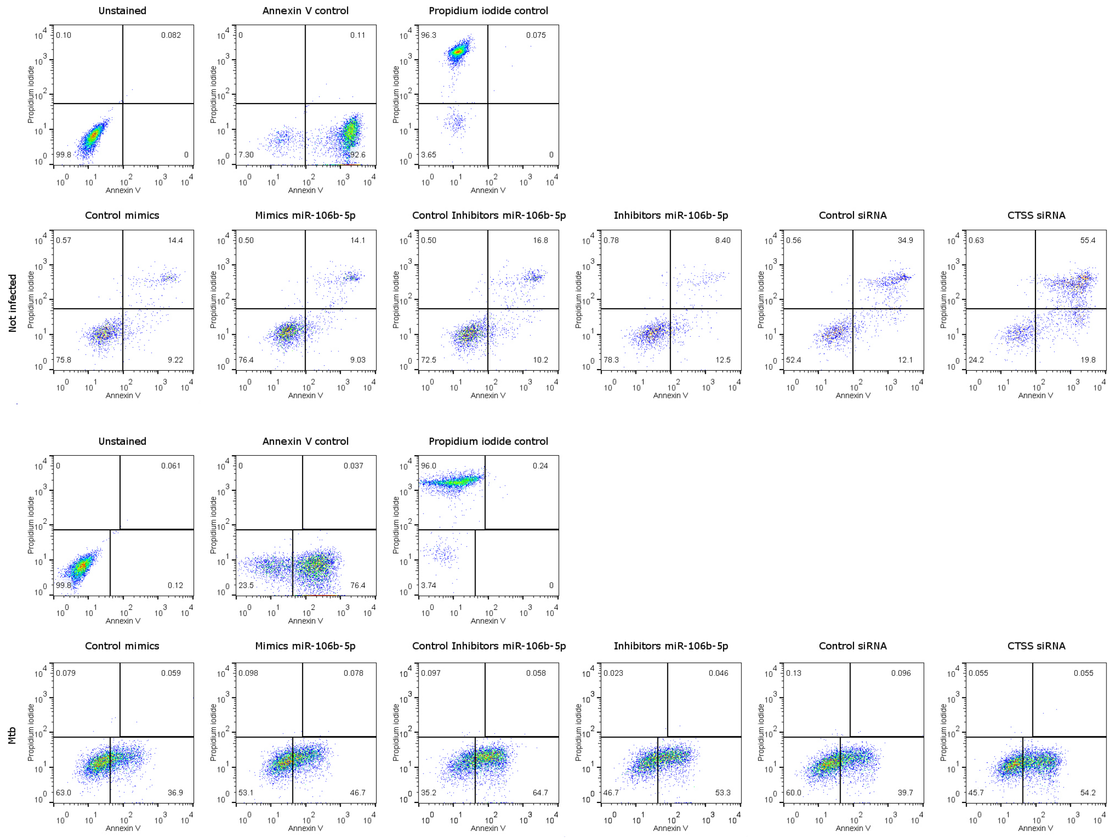

Unstained
Annexin V control
Propidium iodide control
Control mimics
Mimics miR-106b-5p
Control Inhibitors miR-106b-5p
Inhibitors miR-106b-5p
Control siRNA
CTSS siRNA
Not infected
Mtb
Propidium iodide
Annexin V

Figure S2:

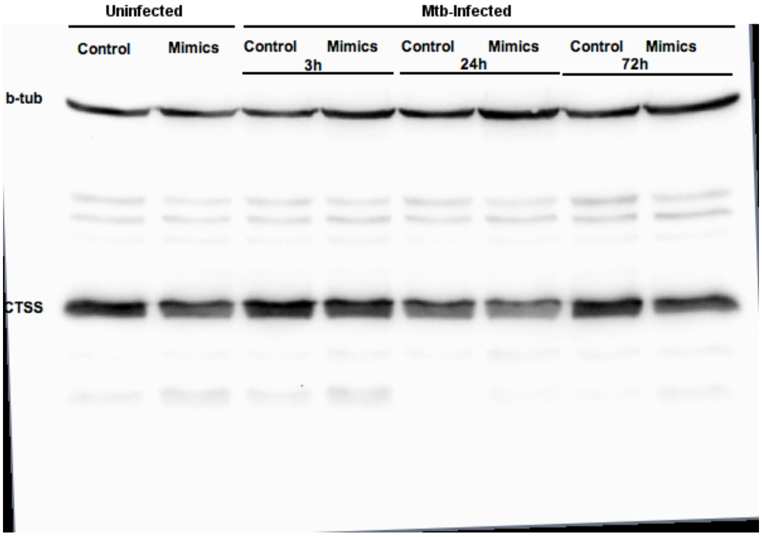
Uninfected
Mtb-Infected
Control
Mimics
Control
Mimics
3h
Control
Mimics
24h
Control
Mimics
72h
b-tub
CTSS

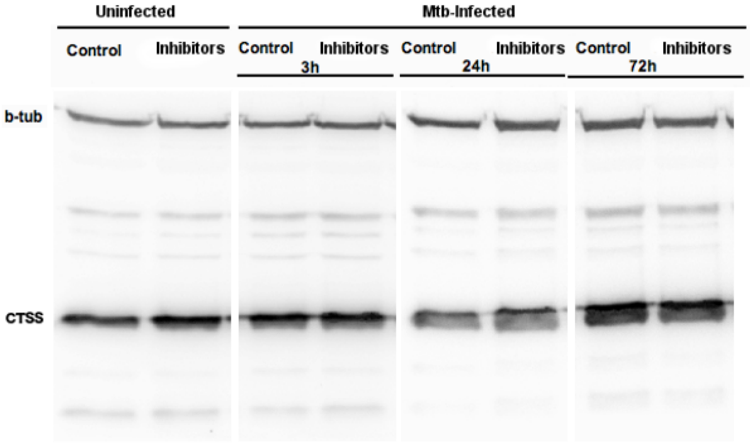
Uninfected
Mtb-Infected
Control
Inhibitors
Control
Inhibitors
3h
Control
Inhibitors
24h
Control
Inhibitors
72h
b-tub
CTSS
